# A specific reverse complement sequence for distinguishing *Brucella canis* from other *Brucella* species

**DOI:** 10.3389/fvets.2022.983482

**Published:** 2022-11-04

**Authors:** Yin-Bo Ye, Jiang-Hua Yang, Dong-Liang Li, Li-Hua Hao, Zhao Zhang, Si-Yao Mei, Huan Zhang, Fang-Yuan Du, Li-Hui Yv, Bao-Shan Liu, Ze-Liang Chen

**Affiliations:** ^1^Key Laboratory of Livestock Infectious Diseases, Ministry of Education, Shenyang Agricultural University, Shenyang, China; ^2^Beijing Animal Disease Prevention and Control Center, Beijing, China; ^3^Division of the Standards, China Institute of Veterinary Drug Control, Beijing, China

**Keywords:** canine brucellosis, *B. canis*, reverse complementary sequence, canine, specific

## Abstract

Canine brucellosis is primarily caused by *Brucella canis*, but other *Brucella* species can also cause the disease. Identifying sequences specific to *B. canis* and establishing PCR assays that can distinguish between *B. canis* and other *Brucella* species is essential to determine the etiology of canine brucellosis and the source of infection and to achieve effective control. We analyzed the gaps and SNPs of genomes I and II from *B. canis* strain RM6/66 and *B. melitensis* strain 16M using the Mauve genome alignment software, and the specificity of each of these differential regions was analyzed by BLAST. A 132 bp specific sequence was found between the DK60_915 (glycosyl hydrolase 108 family protein) and DK60_917 (aldose 1-epimerase) loci in *B. canis* chromosome 1. Further comparative analysis revealed that this is a reverse complement sequence between *B. canis* and other *Brucella* species. Then, three primers were designed based on the sequence that could detect *B. canis* with a 310 bp amplification product or other *Brucella* species with a 413 bp product. The PCR based on these primers had reasonable specificity and a sensitivity of 100 copies of *Brucella* DNA. The detection results for the blood samples of the aborted dogs showed a favorable accordance with the Bruce-ladder multiplex PCR assay. In conclusion, we found a specific reverse complement sequence between *B. canis* and other *Brucella* and developed a PCR method that allows a more comprehensive identification of the pathogen involved in canine brucellosis. These findings provide an effective means for preventing and controlling brucellosis.

## Introduction

Brucellosis is a zoonotic disease caused by *Brucella* species. The number of species in this genus has been gradually expanded by the discovery of strains from wildlife animal species, such as amphibians and fish (1). Four major pathogenic *Brucella* species causing disease in humans are *Brucella abortus* (cattle, buffalo), *Brucella melitensis* (goats, sheep, camels), *Brucella suis* (pigs), and *Brucella canis* (dogs) (2). Currently, more than 500 000 new brucellosis cases are annually diagnosed worldwide (3).

Dogs come into frequent contact with people and can be infected with *Brucella*. The main pathogen in dogs is *B. canis*, but other *Brucella* species can also cause infection or disease (4–6). *B. canis* can be transmitted to humans by infected dogs or their secretions. Unlike other *Brucella* species, the infection symptoms of *B. canis* are absent or mild (7). However, endocarditis or meningitis may develop in some cases (8). Of the other *Brucella* species, *B. abortus* and *B. melitensis* are more pathogenic to humans. Thus, infection of humans by *Brucella* from dogs remains a concern, especially considering that the number of dogs used as pets is significantly increasing.

Isolation from culture is the most accurate form of brucellosis detection, and species identification is conducted by the amino sugar quinovosamine assay (9). However, these methods are time-consuming and need to be performed in a biosafety level III laboratory. Furthermore, the effectiveness is affected by the bacterial load levels in the blood of the infected animal (10). On the contrary, molecular biological detection technologies are safe, reliable, highly sensitive, and strongly specific and require a simple operation. Accordingly, they have been popularized and applied for the detection of *Brucella* (11, 12).

At present, there are a variety of PCR methods for detecting *B. canis* (13, 14), such as the Bruce-ladder (14) and multilocus variable-number tandem-repeat (VNTR) analysis (MLVA) procedures (15). However, most of them distinguish *B. canis* by multiple amplification products; hence, significant efforts are needed to recognize the amplified bands of each *Brucella* species. More importantly, they need high-quality genomic DNA in great concentrations, limiting their use for clinical specimens (16). In 2014, Kang established a PCR method for the specific detection of *B. canis* based on a 12 bp deletion in the BCAN_B0548 region (530056 site) of chromosome II in *B. canis* ATCC 23365 (16). However, it cannot detect other *Brucella* species and probably omits brucellosis caused by other *Brucella* species (17). Using this diagnostic method to test dogs and their owners for *Brucella* may overlook other *Brucella* species; therefore, it is essential to establish a test that can detect both *B. canis* and other *Brucella* species, that can be used to make inferences about the origin of *Brucella* infection in dogs and their owners, and that can be used for prevention and control treatment.

In this study, a specific 132 bp sequence of *B. canis* was found by comparing the genomes of *B. canis* and *B. melitensis* and conducting BLAST alignment of the single nucleotide polymorphisms (SNPs) and indels in GeneBank. A reverse complementary was found between the sequence in *B. canis* and that in other *Brucella* species. Based on this sequence, we developed a multiplex PCR with three primers that can specifically distinguish *B. canis* from other *Brucella* species. This method may be very helpful in preventing and controlling brucellosis and reducing human infection.

## Materials and methods

### Genome alignment of the *B. canis* strain RM6/66 and *B. melitensis* strain 16M

The genome sequences of *B. canis* strain RM6/66 and *B. melitensis biovar* 1. 16M were downloaded from the NCBI database and analyzed by the multiple genome alignment software Mauve 20150226 (The Darling lab at the University of Technology Sydney). The lists of gaps and SNPs from the alignment results of chromosomes 1 and 2 were exported.

### Screening of the specific sequence of *B. canis*

The gaps of both *B. canis* and *B. melitensis* genomes were compared using BLAST for the specific differential sequences of *B. canis*. The candidate differential sequences were aligned by the DNAMAN 7 software (LynnonBiosoft, CA, USA).

### Primer design

Based on the candidate differential sequences, primers were designed by the Primer-BLAST program on the NCBI website to distinguish *B. canis* from other *Brucella* species. Appropriate adjustments were made to obtain more specific primers. The designed primers were synthesized by Sangon Biotech Co., Ltd (Shanghai, China).

### Strains and DNA extraction

All the strains used in this experiment are listed in Table 1. The DNA of *B. abortus* strain 2308, *B. melitensis biovar* 1 strain16M, and *B. canis* strain RM6/66 strains was donated by the China Institute of Veterinary Drug Control. The *B. melitensis* strain M5, *B. abortus* strain A19, and *B. suis* strain S2 were purchased from Tecon Biology Co. Ltd, Xinjiang. The canine Vanguard^®^ Plus 5-CVL and feline Fel-O-Vax^®^ PCT vaccines (Zoetis, NJ, USA) were purchased from a local pet hospital. Other common bacterial strains are preserved in this laboratory. According to the manufacturer's instructions, genomic DNA was extracted using a MiniBEST Bacteria Genomic DNA Extraction Kit (Takara, Dalian, China). Then, the quantity or quality of the extracted DNA was measured with an ultraviolet spectrophotometer, and the copies of the genome were counted online (http://scienceprimer.com/copy-number-calculator-for-realtime-pcr) based on the concentration of DNA and the base number of the *Brucella* genome. The extracted DNA was stored at −20°C for further use. A total of 86 canine blood samples were collected at the pet hospitals in Shenyang. DNA was extracted from the samples using a MiniBEST Whole Blood Genomic DNA Extraction Kit (Takara, Dalian, China) and stored at −20°C. Taq PCR MasterMix was purchased from Vazyme Biotech Co., Ltd, Nanjing, China.

**Table 1:** Bacteria strains tested in this study.

**Bacterial species**		**Strain**		**Source**
*Brucella abortus*		A19		Tecon Biology CO. Ltd
*Brucella suis*		S2		Tecon Biology CO. Ltd
*B.melitensis*		M5		Tecon Biology CO. Ltd
*B. canis*		RM6/66		CVCC
*Brucella abortus*		2308		CVCC
*Brucella suis*		1330		CVCC
*B.melitensis*		16M		CVCC
*Salmonella enteritidis*		CVCC3949		CVCC
*Shigella dysenteriae*		CVCC1881		CVCC
*Pasteurella multocida*		CVCC1676		CVCC
*Streptococcus hemolyticus*		CVCC1886		CVCC
*Clostridium perfringens* type C		CVCC1147		CVCC
*Staphylococcus aureus*		CVCC4098		CVCC
*Proteus mirabillis*		CVCC1969		CVCC
*Candida albicans*		CVCC3597		CVCC
*Streptococcus pyogenes*		CVCC1930		CVCC
*Streptococcus pneumoniae*		CVCC4105		CVCC
*Campylobacter jejuni*		CVCC3883		CVCC
*listeria monocytogenes*		CVCC3763		CVCC
*pseudomonas aeruginosa*		CVCC3795		CVCC
*Escherichia coli*		DH5a		Our laboratory
Vanguard^®^ Plus 5-CVL vaccine	Canine distemper virus	Snyder Hill		Zoetis, USA
	Canine adenovirus type 1			
	Canine adenovirus type 2	Manhattan		
	Canine parainfluenza virus	NL-CPI-5		
	Canine parvovirus	NL-35-D		
	NL-18			
	Leptospira canicola	C-51		
	Leptospira icterohemorrhaiae	NADL		
Fel-O-Vax^®^ PCT vaccine	Feline Rhinotracheitis virus	605		Zoetis, USA
	Feline Calicivirus	255		
	Feline Panleukopenia virus	Cu-4		

CVCC, Chinese veterinary Culture collection center.

### Optimization of the PCR amplification conditions

For the PCR reaction, 10 μl 2× Taq PCR MasterMix, 1 μl of each primer (10 μM), and 6 μl water were mixed in a 200-μl PCR tube. Then, 1 μl of *Brucella* DNA template or 1 μl of distilled water was added as a template or negative control, respectively. The PCR amplification conditions were as follows: pre-denaturation at 94°C for 5 min, 35 cycles at 95°C for 15 s, 60°C for 15 s, and 72°C for 30 s, followed by a final extension at 72°C for 10 min. The PCR amplification products were visualized by electrophoresis on 1.5% agarose gels.

For better amplification conditions, the PCR reaction was optimized by annealing at different temperatures (60°C, 64°C, 68°C, and 72°C). The optimum annealing temperature was determined according to the stray band's existence and the amplified band's brightness.

### Specificity and sensitivity of the assay

The genomic DNA template of *B. canis* strain RM6/66 and *B. melitensis* strain 16M was diluted from 10^3^ to 1 copies/μl with sterilized distilled water. Then, 1 μl diluted DNA was used as the template in the PCR amplification. The PCR reaction contained 10^3^-1 copies of DNA of the *Brucella* gene. Electrophoresis was performed to identify the PCR product. Then, 20 replicates of the DNA at the point of analytical sensitivity were detected in five separate experiments for confirmation.

To verify the specificity, DNAs from other *Brucella* strains and non-*Brucella* bacteria (Table 1) were used as templates for PCR amplification under the optimized PCR conditions. The amplification products were analyzed on a 1.5% electrophoresis gel and observed under ultraviolet light.

### Repeatability test

PCR repeatability experiments were carried out with 10^4^ to 10^2^ copies/μl of the genomic DNA of *B. canis* strain RM6/66 as the template. In the intra-batch repeatability test, the above three samples were tested three times under the same reaction conditions, with three replicates for each sample. In the inter-batch repeatability test, the four samples were tested three times at 1-day intervals, and three replicates were set up for each sample. The amplification products were photographed using the OmegaLumG Gel imaging system (Aplegen Inc, CA, USA) after electrophoresis, and the integrated density values of the bands were calculated using ImageJ 1.52p software (NIH, USA). Then, the mean, standard deviation, and coefficient of variation were calculated to evaluate the repeatability of the PCR.

### Detection of the clinical samples

To evaluate the clinical efficacy of the established PCR method, we collected whole blood samples of aborted dogs in Shenyang, China. DNA was extracted from blood samples of 300 aborted dogs using a MiniBEST Bacteria Genomic DNA Extraction Kit. These DNAs were examined using the established PCR, and the results were compared with those of the Bruce-Ladder multiplex PCR assay (14).

## Results

### Genome analysis

The genome sequences of *B. canis* strain RM6/66 (NZ_CP007758.1, NZ_CP007759.1) and *B. melitensis biovar* 1 strain 16M (NZ_CP007763.1, NZ_CP007762.1) were downloaded from the NCBI database. Their chromosome I (NZ_CP007758.1, NZ_CP007763.1) and II (NZ_CP007759.1, NZ_CP007762.1) sequences were analyzed by the multiple genome alignment software Mauve. Compared with those of *B. melitensis* strain 16M, chromosomes I and II of *B. canis* strain RM6/66 had gene rearrangement (Supplementary Figures 1A,B), and the frequency of chromosome II rearrangement was higher than that of chromosome I.

Aligned gaps and SNPs between *B. canis* and *B. melitensis* were exported from the mauve program (listed in Supplementary Table). There were 204 gaps, 231 inserts, and 5143 SNPs in chromosome I, and 140 gaps, 169 inserts, and 3113 SNPs in chromosome II (Table 2; Supplementary Figure 1C), illustrating that chromosome 1 is more diverse than chromosome 2.

**Table 2:** Insertions and deletions in the *B.melitensis* strain 16M genome compared to the *B. canis* strain 6/66 genome.

**Choromosome**	**Gap**		**SNP**	**Base number of the gaps**	
	**Deletions**	**Insertions**		**Deletions**	**Insertions**
I	204	231	5,143	30,895	19,861
II	140	169	3,113	2,247	31,257

### Screening of the specific sequence of *B. canis*

**Figure 1:**
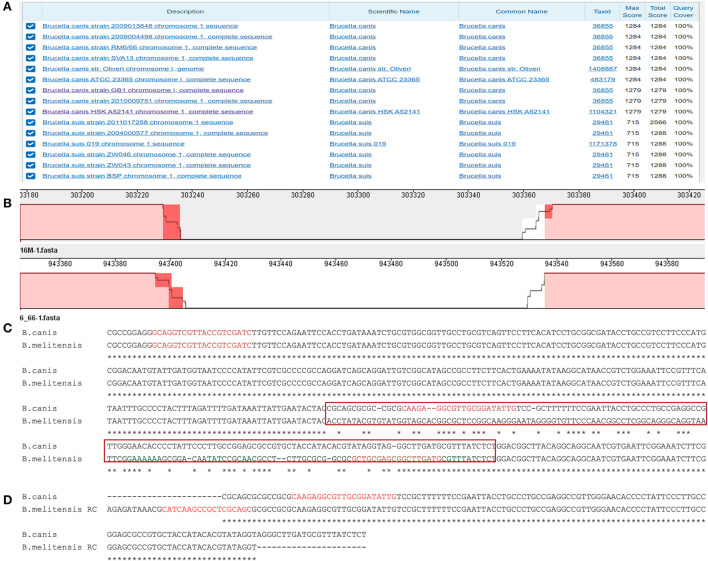
The analysis of the specific sequence in *B. canis*. **(A)** BLAST result of the specific sequence. **(B)** The region of the specific sequence in the genome alignment. **(C)** The alignment of the specific sequences in *B. canis* and the corresponding sequence in *B. melitensis*. The red box represents the sequences in **(D)**. The red sequences represent the designed primers. **(D)** The alignment of the specific sequence in *B. canis* and the corresponding reverse complement sequence in *B. melitensis*.

A BLAST alignment of the gaps and the adjacent sequence of *B. canis* strain RM6/66 showed that a 132 bp gap at the 943403 site on chromosome 1 has reasonable specificity, only having a high similarity and a high score with *B. canis* (Figure 1A). In Mauve, it corresponds to a gap of the same size at the 303367 site on *B. melitensis* chromosome 1 (Figure 1B). Then DNAMAN was used to compare the gaps between the two strains. When the gap with the flanking sequences was aligned, the gap sequence identity between both genomes was low (84.49%; Figure 1C). However, both gap sequences were found to be reversely complementary (Figure 1D). These findings suggest that this sequence is characteristic of *B. canis* and that it can be potentially used for *B. canis* identification. Further analysis showed that it is located in the noncoding region between the DK60_915 (glycosyl hydrolase 108 family protein) and DK60_917 (aldose 1-epimerase) loci.

### Primer design

Three primers (Table 3) were designed based on the differential region. To avoid amplification between two downstream primers, a downstream primer (BSD) used for amplifying other *Brucella* species was selected at the junction of the specific and consensus sequences. The position of each primer is shown in Figure 1C. The sizes of the amplicons of these three primers were 310 bp and 413 bp for *B. canis* and other *Brucella* species, respectively.

**Table 3:** Primers used in this study.

**Name**	**Sequence**	**Position**	**Length**	**Amplicon length**
BSU	GCAGGTCGTTACCGTCGATC	943130-943149 in RM6/66 (302962-302981 in 16M)	20	310 (*B. canis*) 413 (Other *Brucella*)
BCD	CAATATCCGCAACGCCTCTTG	943439-943419 in RM6/66	21	
BSD	CATCAAGCCGCATCGCAGC	(303374-303356 in 16M)	18	

### Optimization of the PCR amplification conditions

**Figure 2:**
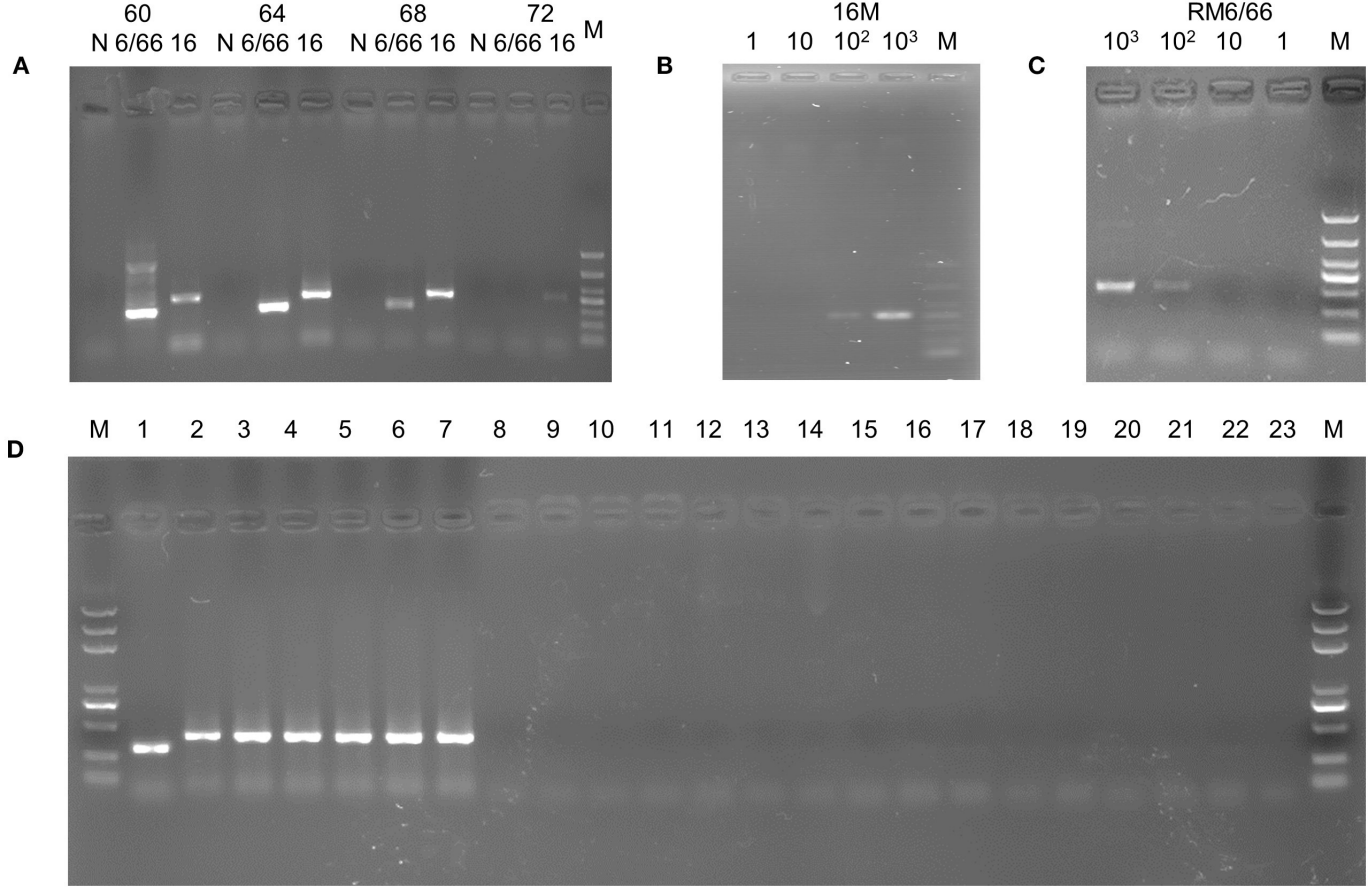
PCR assay based on the specific sequence. **(A)** Annealing temperature optimization. M, DL1000 DNA Marker; 6/66: *B. canis* strain RM6/66; 16: *B.melitensis* strain 16M. **(B)** Sensitivity assay of the *B.melitensis* strain 16M template. M, DL1000 DNA Marker. **(C)** Sensitivity assay of the *B. canis* strain 6/66 template. M, DL1000 DNA Marker. **(D)** Specificity assay. M, DL2000 plus DNA Marker; 1, *B. canis* strian RM6/66; 2, *B. abortus* strain A19; 3, *B. suis* strain S2; 4, *B. melitensis* strain M5; 5, *B. abortus* strain 2308; 6, *B. suis* strain 1330; 7, *B. melitensis* strain 16M; 8, Vanguard^®^ Plus 5-CVL vaccine; 9, Fel-O-Vax^®^ PCT vaccine (Zoetis, USA); 10, *E. coli*; 11, *Salmonella enteritidis*; 12, *Shigella dysenteriae*; 13, *Pasteurella multocida*; 14, *Streptococcus hemolyticus*; 15, *Clostridium perfringens* type C;16, *Staphylococcus aureus*; 17, *Proteus mirabillis*; 18, *Candida albicans*; 19, *Streptococcus pyogenes*; 20, *Streptococcus pneumoniae*; 20, *Campylobacter jejuni*; 21, *Listeria monocytogenes*; 22, *Pseudomonas aeruginosa*; 23, negative control.

To optimize amplification, different annealing temperatures were tested. PCR bands were visible at annealing temperatures ranging from 60°C to 68°C (Figure 2A). However, both amplification products using *B. canis* and *B. melitensis* as templates resulted in bright bands and no evident spurious bands when a 64°C temperature was used. Therefore, 64°C was used as the annealing temperature in the subsequent specificity and sensitivity tests.

### The sensitivity of the assay

To determine the sensitivity of the PCR method, we performed gradient dilution of the DNA from *B. canis* strain RM6/66 and *B. melitensis* strain 16M. The reaction solutions with 10^3^-10^2^ copies of DNA resulted in PCR amplification (Figures 2B,C), indicating that the established PCR assay could detect a minimum of 100 copies of *Brucella* DNA. In the subsequent repetitive detection of DNA samples at sensitive sites, all samples were amplified with visible bands of interest (100%).

### The specificity of the assay

Using DNA of *B. canis* strain RM6/66, *B. abortus* strain A19, *B. abortus* wild type 2308, *B. suis* strain S2, *B. suis* strian 1330, *B. melitensis* strain M5, *B. melitensis* strain 16M, and other non-*Brucella* bacteria (Table 1) as a template, the PCR assay was conducted with the optimal annealing temperature (64°C). The amplicons of *B. canis* DNA and other *Brucella* DNA appeared as distinct 310 bp and 413 bp bands on agarose gels, respectively (Figure 2D). At the same time, no band was amplified when non-*Brucella* DNA was used as a template. These results suggest excellent specificity of the established PCR, demonstrating that the method can detect *Brucella* and distinguish *B. canis* from other *Brucella* species.

### Repeatability test results

DNA from three different dilutions of *B. canis* strain RM6/66 was used to perform intra- and inter-batch reproducibility tests. The integrated gray values of the 400 bp band in the marker and amplification product band were measured using ImageJ software. The number of amplified bands was expressed by the grayscale ratio of amplified bands to 400 bp bands. The mean, standard deviation, and coefficient of variation of three repeated measurements were calculated (Table 4). The coefficients of variation of both intra- and inter-batch reproducibility tests were lower than 10%, indicating that the established PCR method was reproducible.

**Table 4:** The repeatability test of the developed PCR.

**Copies of DNA**	***n***	**Intra-batch repeatability assay**		**Inter-batch repeatability assay**	
		**X ± SD**	**CV(%)**	**X ± SD**	**CV(%)**
10^4^	3	5.125 ± 0.235	4.6	4.651 ± 0.236	5.1
10^3^	3	3.429 ± 0.275	8	3.088 ± 0.302	9.8
10^2^	3	1.229 ± 0.101	8.2	1.368 ± 0.046	3.4

### Detection of the clinical samples

To validate the efficacy of the PCR assay, the DNA of blood samples from the aborted dogs was tested by the developed PCR and the Bruce-ladder multiplex PCR assay (14). The developed PCR detected 22 positive samples with a prevalence of 7.33% (22/300). The prevalence rate for *B. canis* and other *Brucella* was 6.33% (19/300) and 1.00% (3/300), respectively (Table 5). The Bruce-ladder multiplex PCR assay detected 21 positive samples with a prevalence rate of 7.0% (21/300), in which the prevalence for *B. canis, B. melitensis*, and *B. abortus* was 6.0% (18/300), 0.67% (2/300), and 0.33% (1/300), respectively (Table 5). The accordance of the two methods for *B. canis* and other *Brucella* was 94.7% (18/19) and 100% (3/3), respectively. There was no statistical difference between the two methods. These results indicated that the developed assay accurately distinguishes *B. canis* from other *Brucella* species.

**Table 5:** Detection of the blood samples.

**The developed PCR**	**The Bruce-ladder multiplex PCR assay**				**Total**
	***B. canis***	***B. melitensis***	***B. abortus***	**Negative**	
*B. canis*	18	0	0	1	19
Other *Brucella*	0	2	1	0	3
Negative	0	0	0	278	278
Total	18	2	1	279	300

## Discussion

The aim of this study was to find a unique sequence to distinguish between *B. canis* and other *Brucella* species for rapidly detecting canine brucellosis. The designed PCR based on the different sequences demonstrated good specificity, sensitivity, and repeatability. The assay can not only detect brucellosis but can also distinguish *B. canis* from other types of *Brucella*. As a result, it can be applied to the diagnosis of canine brucellosis.

With the development of sequencing, databases, and networking technologies, a vast amount of genomic and sequence data is now available at individual terminals. Making good use of these data has become essential to improving research efficiency. Desktop or web-based bioinformatics software addresses this issue. Multiple Genome Alignment Software Mauve can quickly analyze rearrangements, insertions, deletions, and changes between genome sequences and determine the differences between genes from a macro and micro perspective (18, 19). BLAST is a general alignment procedure that compares sequences similar to target sequences in Genebank databases to elucidate the specificity of target sequences (20). Primer-Blast is a procedure for primer design and comparison that can effectively analyze the specificity of primers (21). The combined use of this software can effectively improve the specificity and success rate of PCR assays, which are widely used (22). In this study, we went through that process and satisfactory results were obtained. First, we found a 132 bp reverse complement sequence between *B. canis* and other *Brucella* species.

Molecular biological detection techniques for distinguishing *Brucella* species such as AMOS-PCR (23, 24) and Bruce-ladder (14, 25) have been established. However, it takes a long time to recognize each *Brucella* species' amplicons. Furthermore, these techniques require high-quality genomic DNA in great concentrations, which limits their use for clinical specimens (16). A PCR method for the specific detection of *B. canis* was established in 2014 based on the 12 bp deletion of chromosome II in *B. canis* ATCC 23365, which had a detection limit of 3 × 10^3^ colony-forming units (16). However, brucellosis in dogs can be caused by other *Brucella* species (4–6); therefore, infection by these species may pass unidentified by this PCR method. This study established a multiplex PCR using three primers for the specific sequence to simultaneously detect *B. canis* and other Brucella species without amplifying other non-*Brucella* bacteria. Its detection limit was 100 copies of the *Brucella* genome, which is higher than that of the abovementioned PCR method. In detecting the blood samples of the aborted dogs, the developed PCR assay demonstrated favorable accordance with the Bruce-ladder multiplex PCR assay.

*B. canis* is the main pathogen causing canine brucellosis. However, other common *Brucella* species, such as *B. suis, B. abortus*, and *B. melitensis*, can also cause it (17). Unlike *B. canis*, they are highly pathogenic to humans. Therefore, people that have had contact with infected dogs are at an increased risk. Consequently, it is necessary to strengthen the diagnosis of brucellosis. The method established in this study can detect the causative agent of canine brucellosis directly, quickly, and comprehensively. However, drawbacks still exist. This PCR cannot distinguish infection caused by a common vaccine strain, and additional tests must be performed to rule this out.

## Conclusion

Here, we found a specific reverse complement sequence between *B. canis* and other *Brucella* species. A multiplex PCR detection method was developed to distinguish *Brucella* species, which can be used for the prevention and control of canine brucellosis and to reduce human brucellosis.

## Data availability statement

The original contributions presented in the study are included in the article/Supplementary material, further inquiries can be directed to the corresponding authors.

## Ethics statement

The animal study was reviewed and approved by Institutional Animal Care and Use Committee of Shenyang Agricultural University. Written informed consent was obtained from the owners for the participation of their animals in this study.

## Author contributions

B-SL conceived the study. Z-LC directed the study. D-LL, L-HY, and L-HH analyzed the data. Y-BY, J-HY, ZZ, and S-YM performed the PCR assays. HZ and F-YD wrote the manuscript. All authors contributed to the article and approved the submitted version.

## Funding

This study was supported by the State Key Program of National Natural Science of China [U1808202], NSFC International (regional) cooperation and exchange program [31961143024], and Major science and technology projects of Inner Mongolia of China.

## Conflict of interest

The authors declare that the research was conducted in the absence of any commercial or financial relationships that could be construed as a potential conflict of interest.

## Publisher's note

All claims expressed in this article are solely those of the authors and do not necessarily represent those of their affiliated organizations, or those of the publisher, the editors and the reviewers. Any product that may be evaluated in this article, or claim that may be made by its manufacturer, is not guaranteed or endorsed by the publisher.
