# Supplementary figures and images for: A specific reverse complement sequence for distinguishing Brucella canis from other Brucella species
Source: Front Vet Sci. 2022 Nov 4;9:983482. doi: 10.3389/fvets.2022.983482 (PMC9672380; doi:10.3389/fvets.2022.983482)

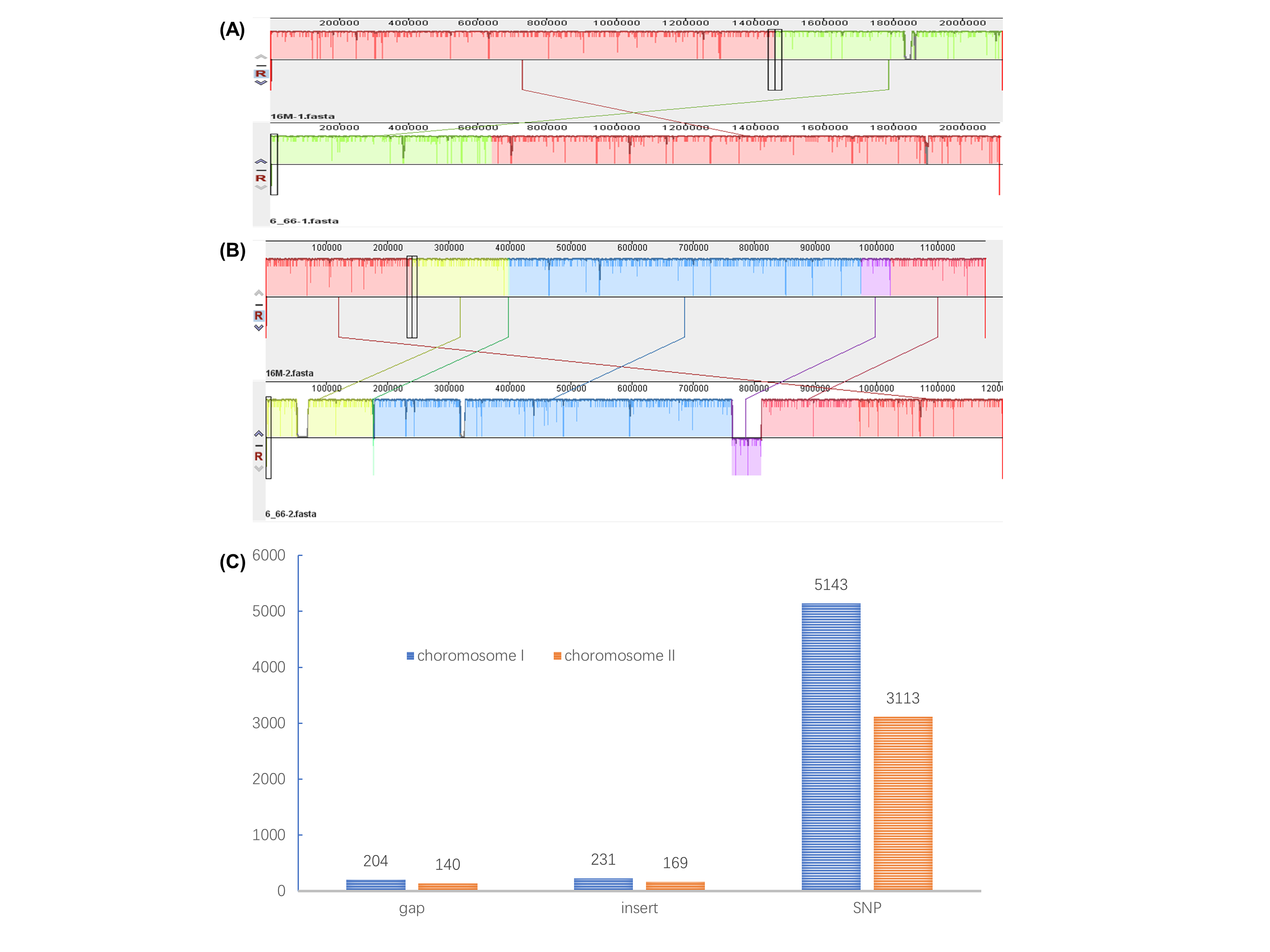

Supplement: Supplementary file 2 [file Image_1.TIF]
